# Evaluation of the Effect of Green Tea Extract on Mouth Bacterial Activity in the Presence of Propylene Glycol

**Published:** 2012-05-28

**Authors:** Abdolhossein Moghbel, Ahmad Farjzadeh, Nasrin Aghel, Homaun Agheli, Nafiseh Raisi

**Affiliations:** 1 Department of Pharmaceutics, School of Pharmacy, Ahvaz Jundishapur University of Medical Sciences, Ahvaz, IR Iran; 2 Department of Microbiology, School of Medicine, Ahvaz Jundishapur University of Medical Sciences, Ahvaz, IR Iran; 3 Department of Pharmacognosy, School of Medicine, Ahvaz Jundishapur University of Medical Sciences, Ahvaz, IR Iran; 4 Department of Medicinal Chemistry, School of Pharmacy, Ahvaz Jundishapur University of Medical Sciences, Ahvaz, IR Iran

**Keywords:** Green Tea Extract, Propylene Glycol, Bacterial Infection, Mouthwash, Tooth, Halitosis

## Abstract

**Background:**

Compounds present in green tea have proved to inhibit the growth and activity of bacteria associated with infections.

**Objectives:**

To assess the effects of green tea leaves extract in presence of propylene glycol on the aerobic mouth bacteria load.

**Materials and Methods:**

Saliva of 25 volunteer girl students aging 20-25 years were selected and evaluated by a mouthwash sample containing 1% tannin, as the most effective antibacterial complex in green tea. Comparative studies were also conducted between green tea mouthwashes containing 1% tannin and a similar sample with 10% propylene glycol added during extraction. This comparison was applied for a chlorhexidine 0.2% sample as a chemical mouthwash brand, too.

**Results:**

There was a meaningful difference between the green tea mouthwashes containing 10% propylene glycol and the simple green tea extract (*P* < 0.05). Significant difference was also seen between the herbal and chemical mouthwashes (*P* < 0.05). The extract 1% tannin containing 10% propylene glycol reduced the aerobic mouth bacterial load of the student salvia about 64 percent. The pH monotonousness in different days and temperatures approved the stability of tannin in liquid water medium.

**Conclusions:**

Using green tea extract as a herbal mouthwash is safe and harmless specially for children and pregnant women. This result led us to suppose that green tea may prevent plaque formation on teeth, coming over halitosis due to mouth infection, too. These effects need to be approved in an in vivo trial as a second study.

## 1. Background

Plant extracts have been widely used as topical and oral applications for disease treatment. Examples of these include ginkgo biloba, echinacea, ginseng, grape seed, green tea, lemon, lavender, rosemary, thuja, sara, allantoin, fever wort blood root, apache plume, papaya and tragacanth (1). Black tea is the second most commonly drank liquid on the earth after water. Green tea camellia sinensis which is not fermented at all during drying process (2) has numerous medicinal benefits mainly due to its antibacterial and antioxidant properties. Tea is native to china but then spread to India and Japan, then to Europe, Russia and finally Iran. A short list of phenolic phytochemicals with promising properties to benefit human health includes a group of polyphenol compounds called catechins, found in green tea (3-5). Compounds present in both green and oolong teas have been studied on dental caries in a simple form and individually (6, 7). Animals and humans given tea compounds in their drinking water develop fewer dental caries and less plaque formation than those drinking plain water (8-11). Drinking green tea may also help prevent sore throats and colds, since it helps fight the bacteria harboring in the throat (12), another reason that encouraged us to work on Iranian green tea other than its healing effect on burn and wound, which was also in the area of our studies (13, 14), was a traditional belief for its masking the bad breath.

## 2. Objectives

The specific aim of this study was to determine the influence of propylene glycol (PG) as a co-solvent, on the potency of green tea extract as a mouthwash, on the staphylococcus aurous strains and Neisseria species of the mouth microbial load. Also, to demonstrate a comparative assessment between the herbal green tea extract containing 10% PG and a chemical brand mouthwash in decreasing mouth bacterial contamination.

## 3. Materials and Methods

### 3.1. Leaves Drying Method and Preparation of Dried Extract Powder

Fresh leaves from field of Lahijan (a city in the North of Iran) were spread out in the hot air to wither. Once they became soft and pliable, they were traditionally pan-fried in woks, after being identified scientifically by the pharmacognosy department, a voucher specimen was deposited at the herbarium of the faculty. In the final step, the leaves were dried by firing as far as the natural fragrances and flavors stabilized, and the leaves kept their green color. The dried leaves were powdered mechanically, and then to obtain the extract, 200 g of 18 mesh powders was added to1000 ml of deionized water at 70-80°C for 30 min. The extract was filtered, by a cloth filter, and concentrated under a low pressure condition installed in a laminar air flow (FARPAJOO-Fee 2120-2) and finally lyophilized (-40^°C^, 0.03 Tor) by a freeze dryer (ZIRBUS Za co-5, Germany) for about 6 hours (6, 15).

### 3.2. Tannin Assay, Validation and Preparation of Calibration Curve

Stock solutions of standard tannic acid were prepared by Rasheed A. *et al*. and Waterman PG *et al*. methods (6, 16). This process was repeated for three or six consecutive days to find the inter–day and intra – day variations. The absorption level was measured specterophotometrically (Shimadzu UV/visible spectrophotometer 2100; Japan) at 760 nm, the mean data (n=10) were used to prepare the calibration curve. The tannin concentration of each sample (mg/100ml) was calculated by regression equation obtained from calibration curve (Y = 0.7051x + 0.0021 and R = 0.998).

### 3.3. Preparation of Equivalent Tannin From Green Tea Powder Extract

Sample containing 1% tannin was prepared by equivalent amount of 16.05 mg of dried green tea powder, yielded from 100mg dried extract (16). The sample was poured into a 100 ml stoppered bottle which had been sterilized by an autoclave at 121^°c^, 15 lb. pressure for 20 min. Volume of the sample was addded to 100 ml of deionized water and finally, filtered through a 0.45 micron membrane filter to be sterilized.

### 3.4. Antimicrobial Evaluation of Green Tea 1% Extract

The target populations of 25 girl students aging 20-25 years were asked to wash their mouths by 10-15 ml deionized water for one minute at 12 o’clock noon as a base time, after routine brushing their teeth with toothpaste at 10 A.M. Mouth content of each student was poured into a sterile container. Aliquots of 0.01 ml of each sample were diluted in 1ml deionized water under sterile conditions and then an aliquot of each new sample was spread on petri dishes containing blood agar. Plates were incubated inside of a candle jar with 5-7 CO_2_ concentration at 35-37°c for 24 hours and then colonies were counted, separately (17). This process was repeated for the extract containing 1% tannin exactly two hours after washing the mouth with 10-15ml deionized water.

### 3.5. Preparation of Mouthwash Formulations

A quantity of 16.5mg green tea dried extract equivalent to 1% tannin was dissolved in about 70 ml, 50°C deionized water and 20 mg sodium saccharin as a non-sugar sweetener. The final volume was adjusted to 100 ml by deionized water after mixing thoroughly and paper filtering. This formula was also repeated with the extra 10% propylene glycol (Merck-Germany). The antimicrobial activity of PG formulation was compared with the chemical chlorhexidine 0.2% as the above mentioned method for the extract.

### 3.6. Stability Evaluation

In order to examine the stability of green tea extract mouthwash containing 10% PG, the mean of pH values (n = 3) was recorded from 48 hours after formulation (zero time) for 3 months. Also, the recorded amount of tannin remained unchanged during 0-90 days holding at different (30, 45, 60^°C^) temperatures (18).

### 3.7. Statistical Analysis and Validation

Data analysis was carried out by student’s t-test and a one-way analysis of variances (ANOVA) using SPSS software followed by TUKEY post hoc test. Significance level was taken at *P* < 0.05 and results were expressed as mean ± SD. The inter and intra-day variations data for validation of the calibration curve were applied and the concentration of the equivalent dissolved drug was calculated by regression equation obtained from the standard curve.

## 4. Results

The inter and intra – day variations of standard curve and accuracy of the tannic acid assay are shown in the Table 1.

**Table 1:** The inter and intra-day variations data of the standard curve and accuracy results in triplicate and 6 different days for tannic acid absorbance and assay by Folin- Deinis method.

	Inter-Day			Intra-Day		
Concentration mg/100 ml	Absorbance, Mean (n=3) ± SD	CV, %	Accuracy% Mean ± SD	Absorbance, Mean (n=3) ± SD	CV, %	Accuracy, % Mean ± SD
0.1	0.066 ± 0.005	8.66	91.57 ± 8.81	0.066 ± 0.002	3.2	91.57 ± 2.98
0.2	0.163 ± 0.005	3.53	114.33 ± 4.09	0.175 ± 0.004	2.7	110.39 ± 3.55
0.3	0.193 ± 0.005	2.98	90.40 ± 2.72	0.195 ± 0.002	1.7	91.71 ± 1.54
0.4	0.29 ± 0.00	0	102.07 ± 0.00	0.291 ± 0.001	0.5	103.25 ± 2.04
0.5	0.343 ± 0.011	3.36	36.79 ± 3.27	0.353 ± 0.006	1.8	100.40 ± 2.7
0.6	0.423 ± 0.0152	3.60	99.56 ± 3.61	0.421 ± 0.002	0.6	98.91 ± 0.77
0.7	0.493 ± 0.011	2.34	99.52 ± 2.33	0.500 ± 0.004	0.9	101.55 ± 1.01
0.8	0.573 ± 0.005	1.00	101 ± 1.02	0.574 ± 0.001	0.2	101.46 ± 0.30
0.9	0.63 ± 0.00	0	98.94 ± 0.00	0.626 ± 0.005	0.5	98.68 ± 0.55
1	0.706 ± 0.005	0.81	99.92 ± 0.81	0.706 ± 0.001	0.9	99.84 ± 0.19

**Figure 1:**
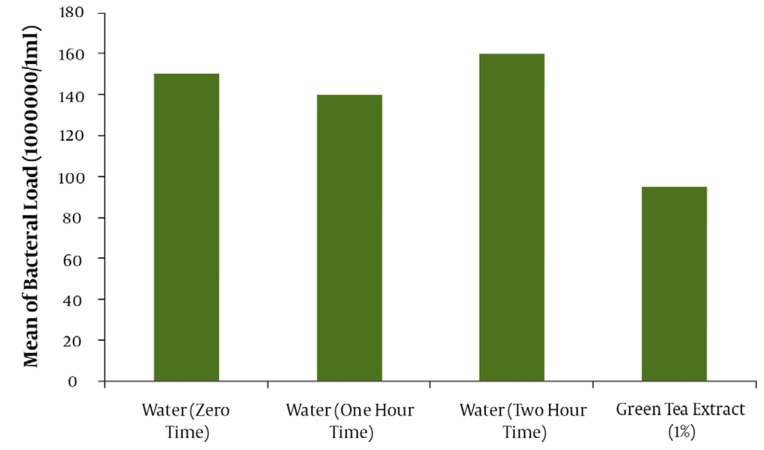
The effect of water and green tea extract containing tannin 1% on mean of mouth bacterial load at different times.

**Figure 2:**
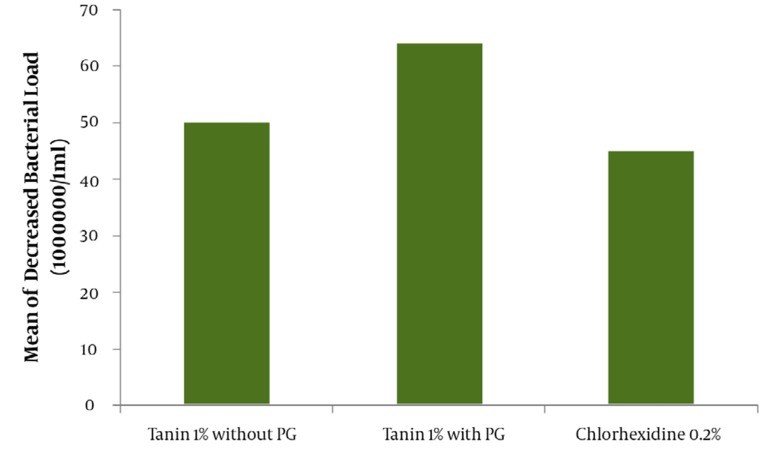
Comparing mouthwashes effect of green tea containing tannin 1% with and without propylene glycol (PG) and chlorhexidine 0.2% on the reduction of aerobic mouth bacterial load (P < 0.05).

The result of mean ± SD for the assay of tannin in green tea extract by Folin- Denis method repeated three consecutive days in triplicate was 6.23 ± 0.19 mg tannin in 100 mg of dried extract powder of green tea (Table 2). The effectiveness of water as a negative control or drug free mouthwash in different times of mouth washing (1 and 2hr. after beginning the trial or zero time) are shown in Figure 1. The comparative results between water as a negative or drug free control and green tea 1% tannin concentration as the most effective antibacterial agent in green tea leaves is also demonstrated in Figure 1 with a significant difference (*P* < 0.05). The comparative results of the mouthwashes containing 1% tannin showed about 64% of bacterial reduction for the mouthwash containing 10% PG and 48% for PG free mouthwash. Evaluation of the effectiveness of the green tea mouthwash 1% tannin containing PG in comparison with the chemical chlorhexidine 0.2% mouthwash showed a higher and significant (*P* < 0.05) reduction on the bacteria (Figure 2). The results of stability evaluation of the product indicated that the amount of tannin remained unchanged during 0 to 90 days holding at different temperatures. The pH evaluation of green tea extract mouthwash showed a constant value (*P* < 0.05) during study (tables and figures not shown). For example the mean value of pH ± SD was about 7.60 ± 0.03. The mean value of tannin 1% remaining unchanged during 0 to 90 days, at different temperatures of 30, 45 and 60°C was calculated 96.08 ± 0.79 percent (*P* < 0.05). The liner specifications such as correlation and regression coefficients, also y-intercept of lines were 0.99, 0.0003 and 2.003, respectively.

**Table 2:** Tannin assay results of green tea or leaves equivalent amount of tannin concentration (n=3)

Sample No.	Weight of green tea extract. total, mg	Absorbance	Tannin concentration, Mg/100ml	Tannin in total extract, %
1	14.28	0.61	0.86	6. 03
2	14.28	0.63	0.89	6.23
3	14.28	0.65	0.91	6.37
Mean ± SD	14.28	0.63 ± 0.02	0.88 ± 0.025	6.23 ± 0.19

## 5. Discussion

Green tea contains flavonoids, tannin, vitamins, fluoride and other mineral salts. Some of antioxidant and antimicrobial agents of green tea could increase the life and efficiency of teeth (19-21). Tannins are biosynthetic materials which have a potent anti-bacterial effect. In recent tooth decay researches, it was mentioned that green and black tea may prevent formation of bacteria in mouth and therefore may reduce construction of plaques on teeth. Also, it was emphasized that the routine consumption of green tea in humans under study might have reduced the intensity of teeth caries (22-24). In our study, which specified the role of propylene glycol as a co-solvent instead of the ethyl alcohol of Yamamoto *et al*. research (24), the results of using green tea mouthwashes showed a good reduction in bacterial colonies. For example, in Yamamoto *et.al* research (24) on green tea in the United State of America, using green tea containing 0.5% tannin with 2% alcohol decreased the different types of *staphylococcus* just about 15%. But, in Iranian alcohol (PG) free tannin 1 % green tea mouthwash we had about 48% and 64% reduction in the bacteria when 10% PG was used. This result shows that Iranian green tea may contain higher amount of tannin or could be better formulated, quantitatively. Besides, using PG instead of harmful ethyl alcohol is not only useful as a co-solvent but also is a powerful chemical stabilizer, as well as intensifying the microbial death with safety for children and pregnant women. The yield value of tannin in 100 mg extract powder of Iranian green tea was calculated 6.23 ± 0.19 mg. The assay of tannin was performed in triplicate with a mean CV% of about 2.6 and accuracy of 100 ± 3.33%.

This result would clarify that choosing the method of Folin–Denis could be accepted as a specific method for this assay. Applying a sample with 1% tannin instead of 0.5 % from Yamamoto’s work (24) could increase the microbial death around twofold and even be extended to threefold when 10% propylene glycol is in charge. Furthermore, another important difference with Yamamoto’s work, other than better efficiency of Iranian green tea antibacterial effect ,was the replacement of propylene glycol with the harmful ethyl alcohol which is a good biological caution, especially in children and pregnant women. Finally, PG could improve the physico-chemical stability and shelf life of the product (18, 24, 25). In This study, a comparison was done between green tea extract and water as a negative control to make sure that water had not influenced the death or removal of the bacteria during rinsing out the mouth with water (Figure 1).This evaluation also was to find the best time for starting the trial with green tea extract. The results of rinsing mouth just with water at different times (e.g. 0.1 and 2 hrs) were analyzed to find out the time interval of coming back the bacteria to about zero time, again. Therefore, the time interval for the bacteria to come back to the same amount of starting time was obtained about 2 hrs (Figure 1). So, the reason of choosing this lag time between the trials or washing the mouth with water 2 hrs before testing with mouthwashes was as mentioned. The comparison of the mouthwash containing 1% tannin with regular water and tannin extract alone (Figure 1) showed a significant difference (*P* < 0.05). The mean reduction of bacterial load of mouthwashes containing 1% tannin with and without propylene glycol (Figure 2) demonstrated a significant difference (*P* < 0.05).The effect of PG and its influence on activity of the extract on bacteria or its role as a co-solvent in the extraction, are quite clear. The comparison of chlorhexidine 0.2% with water (figure not shown) , and tannin 1% containing PG with chlorhexidine 0.2% showed even more significant difference (*P* < 0.05) on the mean reduction of bacterial load (Figure 2). This great difference was because of using 10% polyethylene glycol in the mouthwash and of course it can help the strength. It should be noted that the difference between the PG free 1% tannin of green tea extract and chlorhexidine 0.2% was not significant (*P* > 0.05). Incorporation of the propylene glycol in a water solution as a co-solvent could increase the efficiency of extraction process, especially in the case of oily material of plants (essential oils), in addition to increasing the shelf life and stability of the product. To make sure that the stability of the mouthwash is constant after formulation, the evaluation of green tea mouthwash pH must not show significant change at different times. The result confirmed the constant pH level during three months (*P* > 0.05). Furthermore, the study of chemical stability for green tea mouthwash was followed by calculating the amount of tannin remained unchanged, during zero to 90 days after holding the mouthwash at 30, 45 and 600c temperatures in separate testing conditions (tables and figures not shown). Generally, any dosage form which could have drug activity as much as 90% of the original dose of the formulation, is said to be stable and legally authorized by officials for use(25). Studies showed that green tea, due to its ability to remove the mouth microbial contamination, can eliminate bad breath or halitosis (12). Green tea helps toothpaste and mouthwashes fight viruses by eliminating bacteria. It also helps to prevent plaque formation within gums and teeth (6-10); plaque is another contributor to bad breath. Green tea may prevent bad breath by daily consumption , using it as a mouthwash before and after brushing teeth, or mixing it with the toothpaste products (12).

A herbal mouthwash formulation of Iranian green tea extract containing 1% tannin with 10% propylene glycol could reduce the aerobic mouth bacterial load as much as 45-64% and also, due to this reduction it may prevent plaque formation on teeth and consequently, halitosis. These last claims need to be approved by further study. Replacement and incorporation of propylene glycol in this study for ethyl alcohol in Yamamoto’s research (24) is not only less harmful, especially for children and pregnant women, but it can also influence or increase the strength and antimicrobial effect of green tea extract as well as its stability.

## References

[1] Moghbel A, Hemmati AA, Agheli H, Amraee KH, Rashidi I (2005). The effect of tragacanth mucilage on the healing of full-thickness wound in rabbit.. Arch Iran Med..

[2] David W, Sifton R (2004). PDR for Herbal Medicines..

[3] Chopra D, Simon D (2000). The Chopra Center Herbal Handbook: Forty Natural Prescriptions for Perfect Health..

[4] Moghbel A, Abbaspour H (2010). A study on the factors affecting the compressibility of green tea leaves powder to make a herbal tablet.. Sci Med J..

[5] Mukhtar H, Grupta H, Ahmad N (1998). Inhibition of nuclear transcription factor NFKB by green tea constituent epigallocatechin 3- gallate in human epidermoid carcinoma cells A 431.. J dermatol Sci..

[6] Rasheed A, Haider M (1998). Antibacterial activity of Camellia sinensis extracts against dental caries.. Arch Pharm Res..

[7] Matsumoto M, Minami T, Sasaki H, Sobue S, Hamada S, Ooshima T (1999). Inhibitory effects of oolong tea extract on caries-inducing properties of mutans streptococci.. Caries Res..

[8] Otake S, Makimura M, Kuroki T, Nishihara Y, Hirasawa M (1991). Anticaries effects of polyphenolic compounds from Japanese green tea.. Caries Res..

[9] Ooshima T, Minami T, Aono W, Izumitani A, Sobue S, Fujiwara T (1993). Oolong tea polyphenols inhibit experimental dental caries in SPF rats infected with mutans streptococci.. Caries Res..

[10] Ooshima T, Minami T, Matsumoto M, Fujiwara T, Sobue S, Hamada S (1998). Comparison of the cariostatic effects between regimens to administer oolong tea polyphenols in SPF rats.. Caries Res..

[11] Ooshima T, Minami T, Aono W, Tamura Y, Hamada S (1994). Reduction of dental plaque deposition in humans by oolong tea extract.. Caries Res..

[12] Blasingame J (2009). Green tea prevents bad breath.. http://althealth.mitrasites.com/green-tea-and-bad-breath.html..

[13] Rahimzadeh F, Moghbel A, Kalantar A (2006). formulation of wound healing cream from Iranian green tea extract. A thesis presented for the pharmacy doctorate degree.. Sci Med J..

[14] Rahimzadeh F, Moghbel A (2004). A pilot study of therapeutic effect of water extract (infusion) of Iranian green tea on the healing of full-thickness wound in rabbit. No. 482 approved research project at research deputy.. Sci Med J..

[15] Tory DB (2005). Remington: The Science and Practice of Pharmacy..

[16] Waterman PG, Mole S (1999). Analysis of Phenolic Plant Metabolites..

[17] Baron EJ, Bailley WR, Finegold SM, Bailey & Scott's (1990). Diagnostic Microbiology..

[18] Sweetman SC (2009). Martindale: The Complete Drug Reference..

[19] Berube-Parent S, Pelletier C, Dore J, Tremblay A (2005). Effects of encapsulated green tea and Guarana extracts containing a mixture of epigallocatechin-3-gallate and caffeine on 24 h energy expenditure and fat oxidation in men.. Br J Nutr..

[20] Ferrara L, Montesano D, Senatore A (2001). The distribution of minerals and flavonoids in the tea plant (Camellia sinensis).. Farmaco..

[21] du Toit R, Volsteedt Y, Apostolides Z (2001). Comparison of the antioxidant content of fruits, vegetables and teas measured as vitamin C equivalents.. Toxicology..

[22] Andre B, Barel Paye M, Marc P (2006). Handbook of Cosmetic Science and Technology..

[23] Serafini M, Ghiselli A, Ferro-Luzzi A (1996). In vivo antioxidant effect of green and black tea in man.. Eur J Clin Nutr..

[24] Yamamoto T (1997). Chemistry and Applications of Green Tea..

[25] Lachman L, Lieberman HA, Kanig JL (1986). The Theory and practice of industrial pharmacy..

